# Association Analysis of Peripheral and CSF Biomarkers in Late Mild Cognitive Impairment

**DOI:** 10.3389/fgene.2020.00834

**Published:** 2020-08-12

**Authors:** Tao Zhang, Wei Kong, Shuaiqun Wang, Xiaoyang Mou

**Affiliations:** ^1^College of Information Engineering, Shanghai Maritime University, Shanghai, China; ^2^Department of Biochemistry, Rowan University and Guava Medicine, Glassboro, NJ, United States

**Keywords:** peripheral and CSF biomarkers, blood–brain barrier, late mild cognitive impairment (LMCI), gene set enrichment analysis (GSEA), canonical correlation analysis (CCA), receiver operating characteristic curve (ROC)

## Abstract

Research shows that late mild cognitive impairment (LMCI) has a high risk of turning into Alzheimer’s disease (AD). Due to the invasion of detection methods and physical damage to the patients, it is not a convenient way to diagnose and detect early AD and LMCI by cerebrospinal fluid (CSF) data. So there is an urgent need to find the correlation between peripheral biological data and CSF data in the brain, and to find new diagnostic methods through changes in the peripheral biological data. Studies have shown that during the pathogenesis of LMCI and AD, peripheral immune cells specifically infiltrate into the brain through the blood–brain barrier, causing an imbalance in the brain’s immune response and dysregulating the clearance of Aβ in CSF. Therefore, in this paper, canonical correlation analysis (CCA) algorithm is presented to derive the correlation between peripheral and CSF biomarkers based on LMCI peripheral gene expression data and plasma marker information. Firstly, to explore the influence of the infiltration of peripheral blood immune cells on the brain, the abundance of 28 immune cells were calculated by using the gene set enrichment analysis algorithm of LMCI samples. Then, to identify the correlation between biomarkers inside and outside of the brain, we performed CCA to calculate the relationship between CSF and peripheral biomarkers. Results of CCA showed significant correlations between the variable sets of 8 peripheral biomarkers and the variable sets of CSF biomarkers (at 0.794). Finally, according to Kyoto Encyclopedia of Genes and Genomes and Gene Ontology analysis, it was found that the obtained peripheral biomarkers are involved in many immune-related pathways and functions which can be activated in peripheral blood of LMCI patients. Most related genes enriched in immune-related pathways and functions were up-regulated. Through receiver operating characteristic curve (ROC) analysis, it was also found that FP40/FP42 and type 1 T helper can accurately predict the pathological changes of LMCI (at 0.747).

## Introduction

Mild cognitive impairment (MCI) is a transitional stage in which normal aging develops into dementia (Stephan et al., 2007), but it is also an unstable state. In the follow-up study of MCI patients, the abundance of MCI developing into Alzheimer’s disease (AD) increased year by year. It can be seen that MCI is an early warning signal for the onset of AD (Hansson et al., 2006; Jessen et al., 2014), and the risk of conversion of late mild cognitive impairment (LMCI) to AD is the highest.

Cerebrospinal fluid (CSF) marker analysis is an effective method for diagnosing MCI could be a differentiating marker for the detection of prodromal AD from clinically diagnosed MCI patients (Park et al., 2019). However, due to the invasion of detection methods and physical damage to patients, it is not a convenient way to diagnose and detect early AD and LMCI by CSF. And the study of MCI in Magaki et al. (2007) found that the production of cytokines IL-6, IL-8, and IL-10 increased in peripheral blood, indicating that immune activation is an early phenomenon before AD. Changes in cytokines produced by PBMCs can be detected in MCI and can occur before clinical AD occurs. In the plasma marker study, plasma β amyloid has a certain relationship with β amyloid in the brain, and plasma Aβ measurement can be used as an effective marker to measure Aβ and tau (Risacher et al., 2019). Therefore, in this paper, the easy-to-measure peripheral blood gene data and plasma biomarkers are used to establish an association with CSF markers in the brain of LMCI patients.

To extract the correlation between biological features inside and outside the brain, feature information of biological signals inside and outside the brain needs to be extracted separately. For the calculation of immune cell abundance in peripheral blood, many new calculation methods can greatly enhance our investigation of immune cell subtypes. Among them, the CIBERSORT (Newman et al., 2015) and TIMER (Li et al., 2016) algorithms are based on deconvolution methods, inferring immune cells through gene expression profiling reference matrices and machine learning-based methods. However, these results are obtained from a mixture of simulated samples of different tissues (brain, heart, liver, lung, and tumor tissue), while in blood samples, it is difficult to distinguish (Novershtern et al., 2011; Shoemaker et al., 2012). Bolen et al. (2011) proposed a computational method (from enrichment-related subset prediction, SPEC). The SPEC algorithm is based on gene set enrichment analysis (GSEA; Quesenberry and Colvin, 2001; Subramanian et al., 2005), but it cannot distinguish subpopulations of immune cells in the tagged gene set used to measure immune cells. We obtained a new set of labeled genes through the article (Charoentong et al., 2017) and improved the SPEC algorithm. The advantage of this new algorithm is its robustness, which is insensitive to sample impurities and noise during preparation compared to deconvolution. And the subpopulation of immune cells can also be measured with high resolution.

Biological data sets in CSF can be used to diagnose diseases, but changes in a single indicator in the data set cannot measure the status of the disease. For peripheral biological indicators, a single biological indicator cannot accurately reflect physiological processes. Therefore, to find indicators that can reflect changes in CSF biomarker data in peripheral blood and more comprehensively reflect the physiological process of disease development, the canonical correlation analysis (CCA; Hotelling, 1992) algorithm is used in this paper to calculate the correlation between CSF data sets and peripheral biological data sets. Canonical correlation analysis is a multivariate statistical model that maximizes the correlation between the two composite variables (Kabir et al., 2014). There are more features in the peripheral data, including 28 types of peripheral blood immune cell abundance data and four types of plasma Aβ data. Canonical correlation analysis can more reliably measure the correlation between brain and peripheral biomarkers.

By analyzing the Pearson correlation between CSF data and peripheral data, we obtained that there were significant correlations between peripheral information (including six types of immune cells and two types of Aβ data) and CSF data. Receiver operating characteristic curve (ROC) analysis found that FP40/FP42 (area under the ROC curve, 0.709) and type 1 T helper cell (area under the ROC curve, 0.703) have clinical significance in the diagnosis of the disease. Receiver operating characteristic curve verification found that the combined diagnosis of two biomarkers further improved the accuracy (area under the ROC curve, 0.747). By constructing a protein–protein interaction (PPI) network (Szklarczyk et al., 2015) to find key genes, and performing Gene Ontology (GO) and Kyoto Encyclopedia of Genes and Genomes (KEGG) analysis, the biological processes, and mechanism for the immune-related correlations between peripheral and CSF biomarkers were discovered.

## Materials and Methods

In this section, we describe the measurement of the abundance of peripheral blood immune cells in patients with LMCI and introduce three sources of data. The correlation was calculated and the biological reasons were analyzed.

### Data Sources and Preprocessing

Data used in this study were obtained from the Alzheimer’s Disease Neuroimaging Initiative (ADNI; Weiner and Veitch, 2015) database^1^. The ADNI database was launched in 2003 as a public-private partnership, led by the principal investigator Michael W. Weiner, MD. The ADNI participants have been recruited from more than 50 sites across the United States and Canada. The primary objective of the ADNI has been to test whether serial MRI, PET, other biological markers, and clinical or neuropsychological assessment can be combined to measure the progression of MCI and early AD. Alzheimer’s Disease Neuroimaging Initiative database consists of three parts, including the ADNI 1, the ADNI Grand Opportunities, and the ADNI 2. To date, these three protocols have recruited more than 1500 adults (age range, 55–90 years) to participate in the research, including CN older individuals, persons with early or late MCI, and patients with early AD. The follow-up duration for each study group was specified in the protocols for the ADNI 1, ADNI 2, and ADNI Grand Opportunities. Regional ethics committees of all institutions approved of the study. Written informed consent was obtained from all study participants.

Gene expression profiling from blood samples of ADNI participants was contributed by Bristol-Myers Squibb (BMS) and performed at the BMS laboratories for 811 ADNI participants from the ADNI WGS cohort. The Affymetrix Human Genome U219 Array (Affymetrix^2^, Santa Clara, CA) was used for expression profiling. Peripheral blood samples were collected using PAXgene tubes for RNA analysis. Blood RNA samples from 64 participants did not pass QC and were excluded from further processing. And we identified three questionable subjects from the additional QC steps and removed them. The data we finally downloaded was peripheral blood gene expression data containing 744 samples. The plasma amyloid-beta (Aβ) biomarkers and CSF biomarkers were also obtained from the ADNI database. The plasma Aβ biomarkers contained 305 samples and the CSF biomarkers contained 1250 samples. By screening out samples that existed in all three data and collected three types of biological data in the same year (the samples with missing data were deleted), 36 samples containing three biological data were obtained. The 36 samples were labeled with disease status by using the file “AD Challenge Training Data: Clinical (Updated)” downloaded from the ADNI database.

Finally, the peripheral blood gene expression profile, peripheral blood Aβ biomarkers, and CSF markers contained 36 samples from 20 patients with LMCI and 16 control. The gene expression profile contained 49386 RNAs. The plasma Aβ contained Aβ40 and Aβ42 free in plasma (FP40, FP42), Aβ40, and Aβ42 total in plasma (TP40, TP42). We analyzed two ratios, free plasma Aβ42 to free Aβ40 (FP40: FP42) and total plasma Aβ42 to total Aβ40 (TP40: TP42), as they had been previously shown to correlate with amyloid positivity (Perez-Grijalba et al., 2013, 2019; Fandos et al., 2017; de Rojas et al., 2018). The CSF biomarkers contained Aβ, tau protein (TAU), and phosphorylated tau protein (PTAU).

### Immune Cell Abundance Calculation

In this study, GSEA was applied to calculate the relative abundance of immune cells in peripheral blood. As described in Subramanian et al. (2005) GSEA considers experiments with genomewide expression profiles from samples belonging to two classes, labeled 1 or 2. Genes are ranked based on the correlation between their expression and the class distinction by using any suitable metric. Given an *a priori* defined set of genes S (e.g., genes encoding products in a metabolic pathway, located in the same cytogenetic band, or sharing the same GO category), the goal of GSEA is to determine whether the members of S are randomly distributed throughout L or primarily found at the top or bottom. We calculate an enrichment score (ES) that reflects the degree to which a set S is overrepresented at the extremes (top or bottom) of the entire ranked list L. The score is calculated by walking down the list L, increasing a running-sum statistic when we encounter a gene in S and decreasing it when we encounter genes, not in S. The magnitude of the increment depends on the correlation of the gene with the phenotype. The ES is the maximum deviation from zero encountered in the random walk. Tag gene sets of 28 immune cells were obtained from Charoentong et al. (2017), as *a priori* defined set of genes S. The code for calculating the ES of the custom prior defined set of genes S was obtained from Bolen et al. (2011) (SPEC). We have improved SPEC by replacing the tag gene sets of eight immune cells in the SPEC calculation code with the tag gene sets of 28 immune cells. Download normal and patient peripheral blood gene expression data from the ADNI database, and then GSEA was used to calculate the ESs of 28 immune cell signature genes in the normal and patient sample gene expression data. The ES is the relative abundance of the immune cells in the sample.

### Canonical Correlation Analysis

Canonical correlation analysis is a suitable technique that can establish interrelation between two sets of variables as well as quantify the percentage of variance common to the two sets (Ventura et al., 2011; Kim et al., 2017). Canonical correlation analysis indicates a correlation between two linear combinations of sets of dependent and independent variables as linear combinations of variables useful for predictive or comparative purposes (Akbas and Takma, 2005; Cankaya and Kayaalp, 2007; Sahin et al., 2011). Therefore, the goal of CCA is to find the best linear combination between two multivariate datasets that can maximize the correlation coefficient between them (Malacarne, 2014). Linear combinations of original variables can be defined by canonical variates (Ui and Vi) as follows:

(1) Ui=aiX1+ai2X2+⋯+aipXp

(2) Vi=bi1Y1+bi2Y2+⋯+biqYq

The correlation between Ui and Vi can be defined as canonical correlation. Canonical correlation analysis is repeatedly looking coefficients a and b to maximize the correlation between Ui and Vi. The maximum number of canonical functions that can be extracted equals to the number of variables in the smallest canonical variate (Dattalo, 2014) which is 3 in this study. The first canonical function is derived to maximize the correlation between Ui and Vi variables (Laessig and Duckett, 1979).

Standardized canonical coefficients and loadings were used to evaluate the relative importance of variables in the model (Dattalo, 2014). Standardized coefficients are interpreted similarly to standardized regression coefficients in multiple regressions. Therefore, CCA is used to estimate canonical coefficients (ai1, ai2, …, aip and bi1, bi2, …, biq) when the canonical correlation is at the maximum (Akbas and Takma, 2005). Canonical loading reflects the variance that the observed variable can be shared with canonical variate and interpreted like a factor loading in assessing the relative contribution of each variable to each canonical function (Safari et al., 2013). The result of canonical loading shows the contribution degree of the variable to the variable set. Redundancy index (RI) is proposed to calculate each canonical correlation to determine how much of the variance in one set of variables is accounted by the other set of variables (Sharma, 1996; Safari et al., 2013; Kim et al., 2017). The CCA can use a small number of features to analyze the correlation between the two sets of variables (CSF and peripheral biomarkers). The software SPSS 25.0 for Windows was used for statistical analysis of the data.

## Results

### Immune Cells Abundance Measurement

**FIGURE 1:**
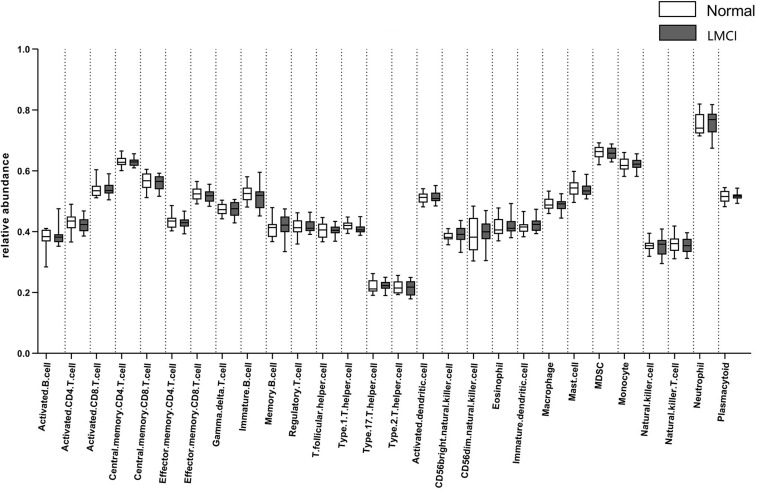
Abundance of 28 peripheral blood immune cells in LMCI and normal samples.

The computational method GSEA was applied to estimate the abundance of 28 kinds of peripheral blood immune cells including 15 kinds of adaptive immune cells and 13 kinds of innate immune cells: activated B cell, immature B cell, memory B cell, activated CD4 T cell, activated CD8 T cell, central memory CD4 T cell, central memory CD8 T cell, effector memory CD4 T cell, effector memory CD8 T cell, gamma delta T cell, regulatory T cell, T follicular helper cell, type 1 T helper cell, type 17 T helper cell, type 2 T helper cell, plasmacytoid, activated dendritic cell, immature dendritic cell, natural killer cell, natural killer T cell, CD56 dim natural killer cell, CD56 bright natural killer cell, eosinophil, macrophage, mast cell, MDSC, natural, and neutrophil. These 28 kinds of immune cells include most types of lymphocytes which produce important cytokines. The abundance of the 28 peripheral blood immune cells for LMCI and normal samples inferred by GSEA were shown in Figure 1.

From Figure 1 we can see, compared with normal samples, the activated CD4 T cell, activated B cell, central memory CD8 T cell, effector memory CD4 T cell, effector memory CD8 T cell, immature B cell, regulatory T cell, T follicular helper cell, type 1 T helper cell, activated dendritic cell, mast cell, MDSC, natural killer T cell appear to be in lower abundance in LMCI samples. However, gamma delta T cell, memory B cell, type 17 T helper cell, type 2 T helper cell, CD56bright natural killer cell, CD56dim natural killer cell, eosinophil, immature dendritic cell, macrophage, monocyte, natural killer cell, neutrophil were increased in LMCI samples.

### Results of Correlation Analysis

At first, to explore the relationship between LMCI and AD, we select proper biomarkers by comparing the changes of CSF biomarkers in LMCI with AD relative to their normal samples respectively (see Supplementary Figure 1). From the Supplementary Figure 1A to Supplementary Figure 1C, we can see that compared with the normal samples, the levels of Aβ are lower, and the levels of TAU and PTAU are higher in the CSF of LMCI. From the Supplementary Figure 1D to Supplementary Figure 1F, the changes in the levels of Aβ, TAU, and PTAU in AD are in the same situation. These results are consistent with the literature in Magaki et al. (2007), McKhann et al. (2011), Dubois et al. (2014) which found that Aβ has a lower level, and TAU and PTAU have a higher level in AD. We can suggest that the LMCI sample has a very high risk of developing AD. Therefore, we selected Aβ, TAU, and PTAU data at baseline as the significant biomarkers of the CSF of LMCI.

Not all peripheral biomarkers are related to CSF biomarkers. To evaluate the correlation between CSF and peripheral biomarkers, the Pearson correlation coefficient (Pearson, 1920) was calculated between CSF and peripheral biomarkers, including three variables in the CSF biomarkers and 34 variables in the peripheral biomarkers. The Pearson correlation coefficients of CSF and peripheral marker variables are shown in Table 1.

**TABLE 1:** Pearson correlation between CSF and peripheral biomarkers.

Immune Cells	CSF data		
	ABETA	TAU	PTAU
Activated B cell	−0.132	−0.111	0.171
Activated CD4 T cell	0.078	0.048	0.062
Activated CD8 T cell	−0.007	−0.135	−0.083
Central memory CD4 T cell	0.290	−0.187	−0.216
Central memory CD8 T cell	−0.003	0.135	0.121
Effector memory CD4 T cell	0.165	−0.181	−0.060
Effector memory CD8 T cell	0.018	−0.221	−0.180
Gamma delta T cell	0.135	−0.042	−0.332*
Immature B cell	0.162	−0.350*	−0.169
Memory B cell	−0.069	0.202	−0.023
Regulatory T cell	−0.051	−0.373*	−0.372*
T follicular helper cell	−0.408*	0.123	0.117
Type 1 T helper cell	0.290	−0.327	−0.401*
Type 17 T helper cell	−0.013	−0.276	−0.107
Type 2 T helper cell	−0.247	−0.011	−0.040
Activated dendritic cell	0.036	0.004	0.133
CD56bright natural killer cell	0.080	0.413*	0.163
CD56dim natural killer cell	−0.230	−0.074	−0.289
Eosinophil	0.231	0.031	−0.178
Immature dendritic cell	−0.046	−0.060	0.209
Macrophage	−0.063	−0.086	0.003
Mast cell	−0.128	−0.105	0.013
MDSC	0.212	−0.078	−0.200
Monocyte	−0.147	−0.116	−0.036
Natural killer cell	0.185	0.166	0.003
Natural killer T cell	−0.151	−0.187	−0.200
Neutrophil	0.116	0.099	0.014
Plasmacytoid	−0.029	0.118	0.182
FP40	−0.175	−0.238	−0.207
TP40	0.000	−0.376*	−0.266
FP42	−0.033	−0.243	−0.328
TP42	0.115	−0.222	−0.282
FP40/FP42	0.074	0.093	0.346*
TP40/TP42	0.093	−0.133	0.031

*At the 0.05 level, the correlation is significant. The meaning of bolded values are significant results obtained in the article.

**FIGURE 2:**
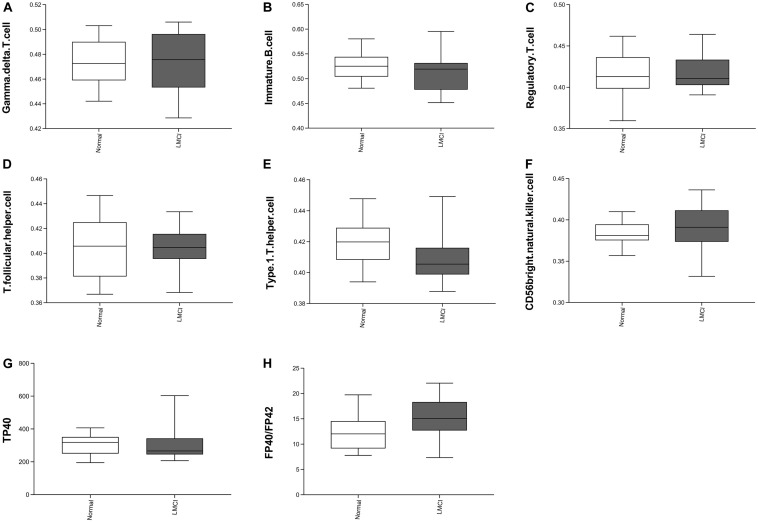
Eight peripheral markers significantly associated with CSF biomarkers. Panel **(A–F)** represents the distribution of the abundance of six immune cells. Panels **(G–H)** represents two peripheral biomarker indicators.

The level of statistical significance was set at α = 0.05. From the Table 1 we can see 8 peripheral biomarkers were significantly associated with CSF biomarkers under significant conditions. The Pearson correlation coefficient between T follicular helper cell and Aβ was −0.408 (α = 0.014 < 0.05); the Pearson correlation coefficient between gamma delta T cell and PTAU was −0.332 (α = 0.048 < 0.05); the Pearson correlation coefficient between immature B cell and TAU was −0.350 (α = 0.036 < 0.05); the Pearson correlation coefficient between regulatory T cell and TAU was −0.373 (α = 0.025 < 0.05); the Pearson correlation coefficient between regulatory T cell and PTAU was −0.372 (α = 0.025 < 0.05); the Pearson correlation coefficient between type 1 T helper cell and PTAU was −0.401 (α = 0.015 < 0.05); the Pearson correlation coefficient between TP40 and TAU was −0.376 (α = 0.024 < 0.05); Pearson correlation coefficient of FP40/FP42 and PTAU was 0.346 (α = 0.038 < 0.05). The levels of 8 peripheral biomarkers in LMCI and normal samples are shown in Figure 2.

Compared with the normal samples, we can see from Figure 2 that the abundances of gamma delta T cell increased slightly; the abundance of immature B cell, regulatory T cell, T follicular cell, and TP40 decreased slightly; the abundance of CD56 bright natural killer cell and FP40/FP42 increased significantly; type 1 T helper cell abundance reduced significantly.

Bivariate correlations among variables of CSF biomarkers and variables of peripheral biomarkers are shown in Table 1. Results showed correlations between variable sets of Aβ, TAU, or PTAU (CSF biomarkers) and variable sets of gamma delta T cell, immature B cell, regulatory T cell, T follicular cell, type 1 T helper cell, CD56bright natural killer cell, TP40, or FP40/FP42(8 of the peripheral biomarkers). As we know that MCI was a complicated brain disease and the occurrence of MCI was accompanied by changes in the levels of multiple CSF biomarkers. One CSF biomarker cannot accurately determine the occurrence of disease. Since the Pearson correlation only could explain the correlation between two variables, it is impossible to simultaneously discover the relationship between the variable set of CSF and peripheral biomarkers. Base on that, in this study, CCA was introduced to explain the relationship between CSF biomarkers and peripheral biomarkers. Table 2 shows direct results for the correlation between the two variable sets, which presented the canonical correlation coefficients and the significance of the research.

**TABLE 2:** Summary of results from canonical correlation analysis.

	Correlation	Eigenvalue	Wilks Statistic	*F*	Sig.
1	**0.794**	1.702	0.131	3.094	0.000
2	**0.709**	1.013	0.354	2.530	0.008
3	0.536	0.404	0.712	1.818	0.133

*The meaning of bolded values are significant results obtained in the article.*

Table 2 showed that two of the 3 confirmed canonical correlations were statistically significant. The first canonical correlation was 0.794. It represented the highest possible correlation between any linear combinations for three CSF biomarkers (U1) and eight peripheral biomarkers (V1) (*p* < 0.01). The second canonical correlation was 0.709. It indicated that the relationship between canonical variates was significant (*p* = 0.008 < 0.01). However, the canonical correlation of correlation 3 was not statistically significant.

Standardized canonical coefficients for pairs of all canonical variables [Ui and Vi in formula (1) and (2)] were shown in Table 3. Canonical variates representing optimal linear combinations of dependent and independent variables were defined by C1. Standardized canonical coefficients of Aβ (0.435 in C1 and −0.493 in C2), TAU (0.867 in C1 and −0.886 in C2), and PTAU (0.339 in C1 and 1.062 in C2) as variables of CSF biomarkers suggested that they contributed importantly to the first and second canonical variate (U1, U2). On the other hand, the first standardized canonical coefficients of immature B cell, regulatory T cell, CD56 bright natural killer cell, gamma delta T cell, TP40 as variables of peripheral biomarkers were −0.392, −0.585, 0.514, −0.423, and −0.456, respectively, indicating that they contributed importantly to the first canonical variate (V1). The second standardized canonical coefficients of FP40/FP42(0.61), T follicular helper cell (0.641), type 1 T helper cell (−0.390), gamma delta T cell (−0.580), and TP40 (0.340) contributed importantly to the second canonical variate (V2).

**TABLE 3:** Standardized canonical correlation coefficients.

Variable	C1	C2	C3
Aβ	**0.435**	**−0.493**	0.914
TAU	**0.867**	**−0.886**	−0.530
PTAU	**0.339**	**1.062**	0.752
FP40/FP42	−0.019	**0.613**	0.657
Immature B cell	**−0.392**	0.171	0.240
Regulatory T cell	**−0.585**	−0.040	−0.247
T follicular helper cell	0.123	**0.641**	−0.573
Type 1 T helper cell	0.158	**−0.390**	0.422
CD56bright natural killer cell	**0.514**	−0.259	0.071
Gamma delta T cell	**−0.423**	**−0.580**	−0.146
TP40	**−0.456**	**0.340**	0.328

*C1,C2,C3:1st,2nd,3rd standardized canonical coefficients. The meaning of bolded values are significant results obtained in the article.*

To find out the key factors in each group of variables, we presented the loadings for the canonical function in Table 4. Canonical loading presents a product-moment correlation between the original variable and its corresponding canonical variate. These values reflect the degree of a variable to be represented by a canonical variate. Canonical loadings for variables of CSF biomarkers suggested that TAU (0.905) and PTAU (0.723) had more effect than Aβ (0.07) to form the first fair for variables of CSF biomarkers (U1). In the second fair for variables of CSF biomarkers (U2), Aβ (−0.561) and PTAU (0.691) were more important factors. On the other hand, canonical loadings for regulatory T cell (−0.595), CD56bright natural killer cell (0.565), TP40 (−0.525), type 1 T helper cell (−0.370), immature B cell (−0.366), and FP40/FP42 (0.290) had stronger effects compared to other factors to form the first fair for variables of peripheral biomarkers (V1). And the FP40/FP42 (0.351), T follicular helper cell (0.305), type 1 T helper cell, CD56bright natural killer cell (−0.326), and gamma delta T (−0.538) cell were more important factors to form the second fair for variables of peripheral biomarkers (V2).

**TABLE 4:** Canonical loadings of original variables with their canonical variables.

	CSF group set				Peripheral group set							
	ABETA	TAU	PTAU		FP40/FP42	Immature B cell	Regulatory T cell	T follicular helper cell	Type 1 T helper cell	CD56bright natural killer cell	Gamma delta T cell	TP40
***U1***	−0.07	**0.905**	**0.723**	***V1***	**0.29**	**−0.366**	**−0.595**	−0.039	−**0.37**	**0.565**	−0.114	**−0.525**
***U2***	**−0.561**	0.012	**0.691**	***V2***	**0.351**	0.073	−0.056	**0.305**	**−0.393**	**−0.326**	**−0.538**	0.072
***U3***	0.825	−0.425	0.029	***V3***	0.52	0.385	−0.241	−0.653	0.254	−0.042	−0.194	0

***U1, U2**, and **U3**, 1st, 2nd, and 3rd CSF variables; **V1**, **V2**, and **V3**, 1st, 2nd, and 3rd peripheral variables; CSF group set: CSF biomarkers; Peripheral group set: Peripheral biomarkers. The meaning of bolded values are significant results obtained in the article.*

Numbers of dimensions explaining the relationships between variable sets were reduced from 11 to 2 by CCA. These results indicated a high correlation between the set of CSF biomarkers (U1) and the set of peripheral biomarkers (V1) (at 0.794). Cross loadings represent correlations between original variables and opposite canonical variables. According to cross-loading results, the first pair of canonical variables TAU (0.718) and PTAU (0.574) provided a relatively strong contribution to canonical variate V1 whereas regulatory T cell (−0.472), CD56bright natural killer cell (0.448), TP40 (−0.417), type 1 T helper cell (−0.294), immature B cell (−0.291), and FP40/FP42 (0.230) highly contributed to U1 (Table 5). The second pair of canonical variables Aβ (−0.398) and PTAU (0.49) provided a greater contribution to canonical variate V2 whereas FP40/FP42 (0.249), T follicular helper cell (0.216), type 1 T helper cell (−0.279), CD56bright natural killer cell (−0.231), and gamma delta T cell made an important contribution to U2 (Table 5).

**TABLE 5:** Cross loadings of original variables with opposite canonical variables.

	CSF group set				Peripheral group set							
	ABETA	TAU	PTAU		FP40/FP42	Immature B cell	Regulatory T cell	T follicular helper cell	Type 1 T helper cell	CD56bright natural killer cell	Gamma delta T cell	TP40
***U1***	−0.055	**0.718**	**0.574**	***V1***	**0.23**	**−0.291**	**−0.472**	−0.031	**−0.294**	**0.448**	**−0**.09	**−0.417**
***U2***	**−0.398**	0.008	**0.49**	***V2***	**0.249**	0.052	−0.04	**0.216**	**−0.279**	**−0.231**	**−0.382**	0.051
***U3***	0.442	−0.228	0.015	***V3***	0.279	0.207	−0.129	−0.35	0.136	−0.023	−0.104	0

***U1, U2** and **U3**, 1^st^, 2^nd^, and 3^rd^ CSF variables;** V1, V2**, and **V3**, 1st, 2nd, and 3rd peripheral variables; CSF group set: CSF biomarkers; Peripheral group set: Peripheral biomarkers. The meaning of bolded values are significant results obtained in the article.*

#### ROC Analysis

**FIGURE 3:**
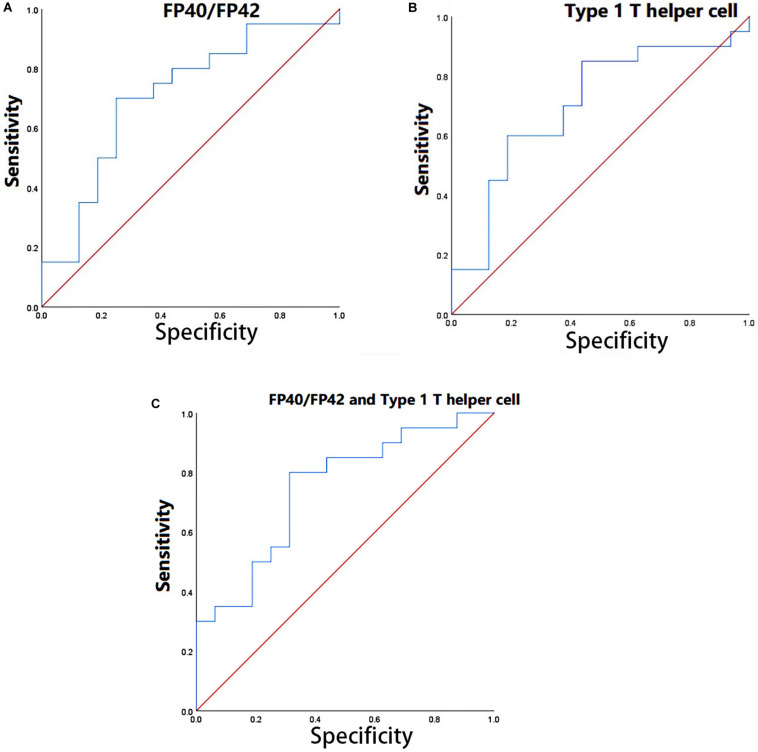
**(A)** ROC analysis in FP40F/FP42. **(B)** ROC analysis in type 1 T helper cell. **(C)** ROC analysis in the combination of FP40/FP42 and type 1 T helper cell.

To verify the practical significance of the correlation between CSF and peripheral biomarkers, we conducted the ROC analysis. Through CCA analysis, variables with a significant correlation between CSF and peripheral biomarkers were found. Receiver operating characteristic curve analysis by using the peripheral biomarkers (type 1 T helper cell, CD56bright natural killer cell, and FP40F/P42 highly contributed to U1 and U2) which highly contributed to V1 and V2 was performed to find peripheral biological indicators with clinical significance for disease classification. Then multiple peripheral biomarkers were used for logistic regression, and ROC analysis of multi-index combined classification was performed. We used SPSS software to draw the ROC curve. The results of the ROC analysis were shown in Figure 3.

Through the ROC analysis, we found that FP40/FP42 (area under the ROC curve, 0.709) and type 1 T helper cell (area under the ROC curve, 0.703) had clinical significance for the diagnosis of the disease (Figures 3A,B). Logistic regression was performed by using two indicators (set the coefficient significance to *p* < 0.1). The coefficients of FP40F/FP42 (*p* = 0.046 < 0.1) and type 1 T helper cell (*p* = 0.093 < 0.1) satisfy the significance. The ROC analysis of the combined diagnosis of the two indicators showed that the combined diagnosis further improved accuracy (the area under the ROC curve, 0.747) (Figure 3C).

### Reconstruction of the PPI Network

The immune-related PPI network was constructed to explore the biological significance of the correlation between CSF and peripheral biomarkers. For the differential analysis of the microarray data used to calculate the abundance of immune cells, differential RNA extraction was performed by using the Limma algorithm. We chose *p*-value < 0.05 as the threshold for screening differentially expressed genes. This process is implemented using the R package ‘Limma’ (Ritchie et al., 2015). A total of 1324 differentially expressed genes (DEGs) were identified, of which 801 were up-regulated and 523 were down-regulated in LMCI. For all the differentially expressed genes, we constructed the PPI network by using the STRING database^3^. The final DEGs PPI network was constructed with 1050 gene nodes and 4547 edges through the STRING v10 data database.

**FIGURE 4:**
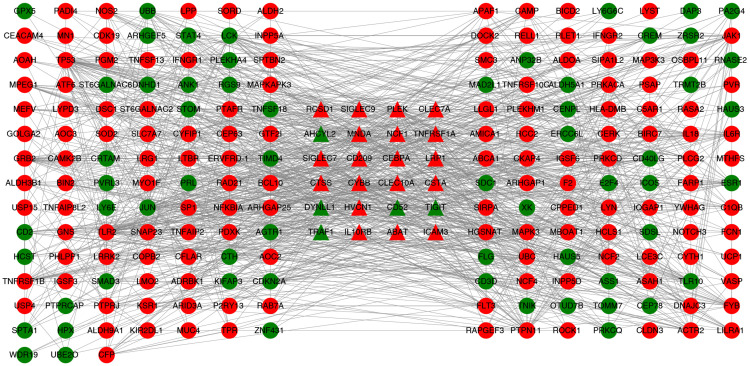
PPI network associated with immune cells. Twenty-four triangular nodes are gene nodes associated with immune cells. Other gene nodes were obtained by screening from PPI networks constructed from differentially expressed genes. Red is up-regulated in gene expression and green is down-regulated in gene expression.

Protein–protein interaction network related to the abundances of immune cells was constructed by retaining genes related to immunity and node genes directly linked to them. In the correlation analysis, six immune cells were significantly correlated with CSF biomarkers. 1324 differentially expressed genes were matched with the tag genes of 6 immune cells, and 26 immune-related differentially expressed genes were obtained. We used 26 immune-related differentially expressed genes to search in the constructed PPI network and found that 1 gene does not exist in the PPI network and 1 gene is an isolated node. Finally, 2 genes were deleted and 24 immune-related differentially expressed genes were retained. In the PPI network, 24 immune-related differentially expressed genes and gene nodes directly connected to them were selected, the immune-related PPI network was constructed by 223 gene nodes and 1123 edges finally. The visualization of the immune-related PPI network was built using Cytoscape v3.6.0 software (Shannon et al., 2003) (Figure 4).

**FIGURE 5:**
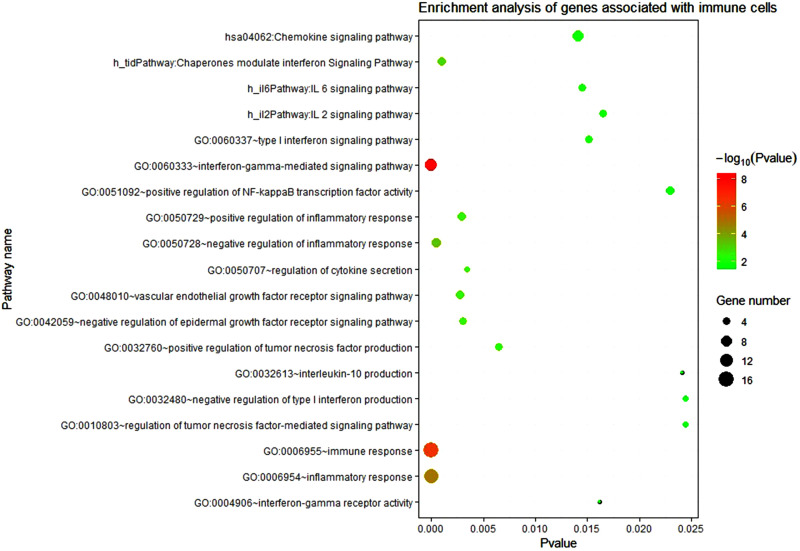
Enrichment analysis of genes associated with immune cells. The circle size represents the number of enriched genes and the color represents the P-value. The vertical axis represents the name of the relevant pathway and biological process, and the horizontal axis represents the percentage of the gene.

Then the 223 significant genes were analyzed by DAVID for GO and KEGG, and we investigated the role of these genes in biological functions and processes (see Figure 5). The GO and KEGG analysis used the online database DAVID v6.8 (Sherman and Lempicki, 2009), and visual display through R software (R Core Team, 2013).

The results of the enrichment analysis showed that these genes were related to the inflammatory response and had a certain relationship with the expression of some cytokines. In the reconstructed PPI network, most genes were up-regulated (a total of 223 genes, 157 genes were significantly up-regulated and 68 genes were significantly down-regulated). Among the 24 immune-related genes, 19 genes were significantly up-regulated and 5 genes were down-regulated. It shows that LMCI is closely related to the systemic inflammatory response. LMCI may show similar physiological processes as AD, such as the recruitment of peripheral immune cells to the brain, and the permeability of blood–brain barrier (BBB). It may be the biological reason for the significant correlation between the LMCI peripheral data and the CSF data.

## Discussion

MCI is a chronic degenerative disease of the nervous system, which refers to a state of cognitive impairment between normal aging and dementia. It appears that memory loss is not commensurate with age, but it has not yet reached the standard of AD. However, patients diagnosed with MCI are at high risk of developing AD. The AD conversion rate is 6–25% per year. In the AD research, many clinical cases have shown that the treatment effect is not good in the middle and advanced stages, so researchers have turned their attention to its early diagnosis and preventive intervention in recent years. There is evidence that peripheral immune cells in AD recognize Aβ and treat it, present it to T cells, and trigger adaptive immunity (Jóźwik et al., 2012; Begum et al., 2014). This indicates that peripheral biomarkers may be potentially associated with AD. The CSF biomarker changes in LMCI patients are very similar to those in AD, so the study of LMCI patients is of great significance for early detection and early intervention of AD. CCA was introduced to explain the relationship between CSF biomarkers and peripheral biomarkers. The results indicated a high correlation between variable sets of CSF biomarkers and variable sets of peripheral biomarkers. It may be due to Aβ transported to the periphery and activated adaptive immunity. Receiver operating characteristic curve analysis found that the diagnostic accuracy of two of the peripheral biomarkers (FP40/FP42 and type 1 T helper cell) for the disease was 0.747. It was found that the systemic immune response plays an important role in the correlation between peripheral biomarkers and CSF biomarkers through KEGG and GO analysis.

About 223 differentially expressed genes related to immunity were used for KEGG and GO analysis. It was found that these genes are closely related to the IL-6 signaling pathway, TNF signaling pathway, IFN-γ signaling pathway, γ-interferon- mediated signaling pathways, tumor necrosis factor-mediated signaling pathway, chemokine signal pathway, inflammatory response function, immune response function, and so on. From the biological analysis we can see that these pathways and functions in KEGG and GO results are related to immunity, inflammation, and cytokines. We also found other biological processes related to the immune response, such as positive and negative regulation of inflammatory response function, type I interferon signaling pathway, tumor necrosis factor-activated receptor activity function, positive regulation of tumor necrosis factor production function, regulation of γ-interferon-mediated signaling pathway. Most genes enriched in these pathways and functions are up-regulated.

The further molecular biological analysis shows that the expression of Aβ in CSF and peripheral is closely related. It has been found in AD studies that Aβ enters peripheral blood to trigger an inflammatory response. PBMCs from AD patients are induced to release pro-inflammatory cytokines such as IL-6, TNF, and IFN-γ (Pellicano et al., 2010). It further promoted the production of amyloid precursor protein and enzymes that cleave it, leading to increased Aβ production (Sutinen et al., 2012). Aβ can also stimulate the pro-inflammatory NF-κB dependent signaling pathway (Kumar et al., 2014). Through regulated transport, peripheral inflammatory markers can cross the BBB and perform neuromodulation (Banks, 2005). INF-γ stimulated the release of CXCL-10, and INF also increased. In AD, CXCL-10 has been found to bind to the chemokine receptor CXC chemokine receptor 3, which is involved in T cell initiation and maintenance of natural killer cells in the body, thereby inducing extracellular signal-regulated kinases Pathways eventually lead to neuronal dysfunction and apoptosis (Nelson and Gruol, 2004; Sui et al., 2006; Cho et al., 2009). Compared with normal samples, the concentrations of peripheral blood cytokines IL-2, IL-6, and epidermal growth factor (EGF) in AD patients were significantly increased (Lai et al., 2017). Physiological changes including inflammatory responses and changes in cytokines were found in AD. Kyoto Encyclopedia of Genes and Genomes and GO analysis of differentially expressed genes in this study also found similar physiological changes in pathways and functions in LMCI patients. Kyoto Encyclopedia of Genes and Genomes and GO analysis of differentially expressed genes in peripheral blood of LMCI patients revealed that most of the genes enriched in cytokine-related signaling pathways were up-regulated. Aβ clearance in the brain mainly depends on peripheral clearance to achieve local lymph node degradation. In LMCI, Aβ in the brain is transported to the peripheral blood and releases pro-inflammatory cytokines, activating immune-related pathways and functions. This indicates that patients with LMCI have shown a systemic inflammatory response similar to AD patients. These results show that peripheral biomarkers can reflect the pathological changes of LMCI in the brain. Peripheral biomarkers may develop new diagnostic methods for LMCI. The research on LMCI will also help to predict AD at an early stage, and also provide an important basis for AD immunotherapy.

## Data Availability Statement

Publicly available datasets were analyzed in this study. This data can be found at ADNI datasets: https://ida.loni.usc.edu/pages/access/studyData.jsp; https://ida.loni.usc.edu/pages/access/geneticData.jsp.

## Ethics Statement

Written informed consent was obtained from the individual(s) for the publication of any potentially identifiable images or data included in this article.

## Author Contributions

WK and TZ conceived the study, participated in its design and coordination, and performed all the molecular biological analyses of the data. TZ carried out the GSEA studies on the LMCI’s gene expression data and drafted the manuscript. SW and TZ performed the pre-processing, GSEA, CCA algorithms, and some statistical analysis of LMCI expression data and plasma marker data. WK helped with data interpretation and manuscript drafting. XM participated in the final data analysis and interpretation. All authors read and approved the final manuscript.

## Conflict of Interest

The authors declare that the research was conducted in the absence of any commercial or financial relationships that could be construed as a potential conflict of interest.

## Footnotes

1 http://adni.loni.usc.edu

2 www.affymetrix.com

3 https://string-db.org/

## Supplementary Material

FIGURE S1 Change of CSF biomarkers in LMCI **(A–C)** and AD **(D–F)**.
