# Supplementary figures and images for: Association Analysis of Peripheral and CSF Biomarkers in Late Mild Cognitive Impairment
Source: Front Genet. 2020 Aug 12;11:834. doi: 10.3389/fgene.2020.00834 (PMC7437457; doi:10.3389/fgene.2020.00834)

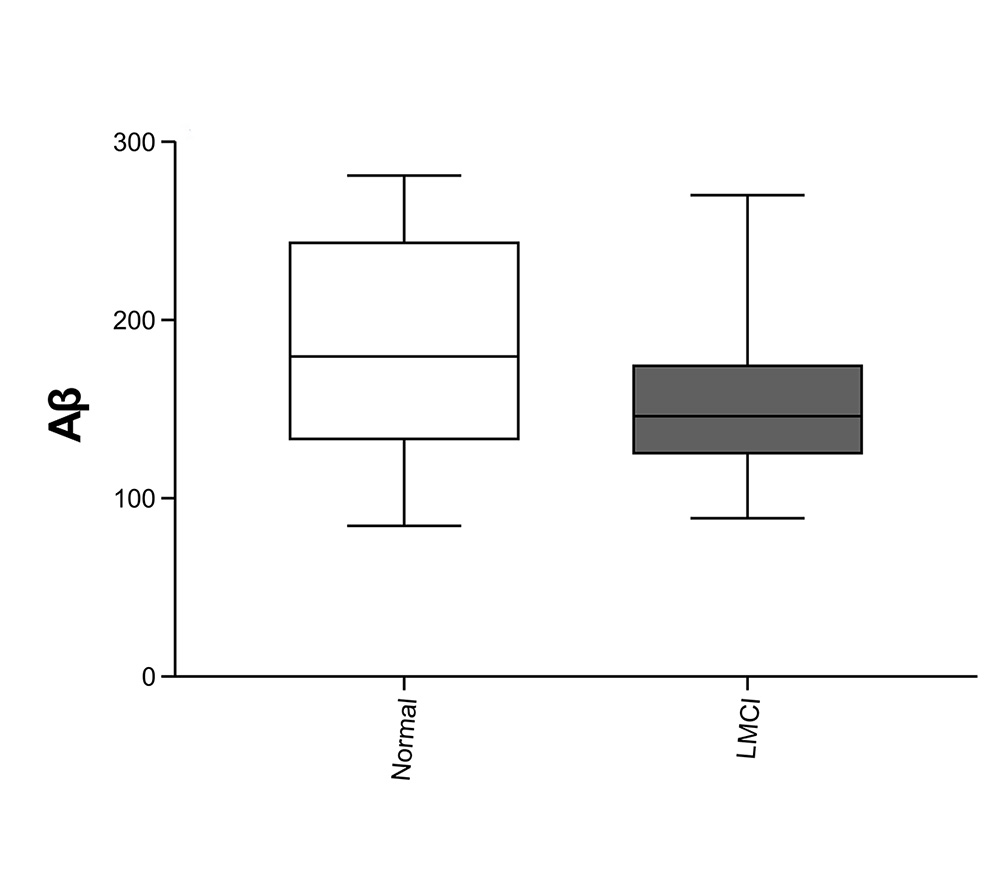

Supplement: FIGURE S1 — Change of CSF biomarkers in LMCI (A–C) and AD (D–F). [file Image_1.JPEG]

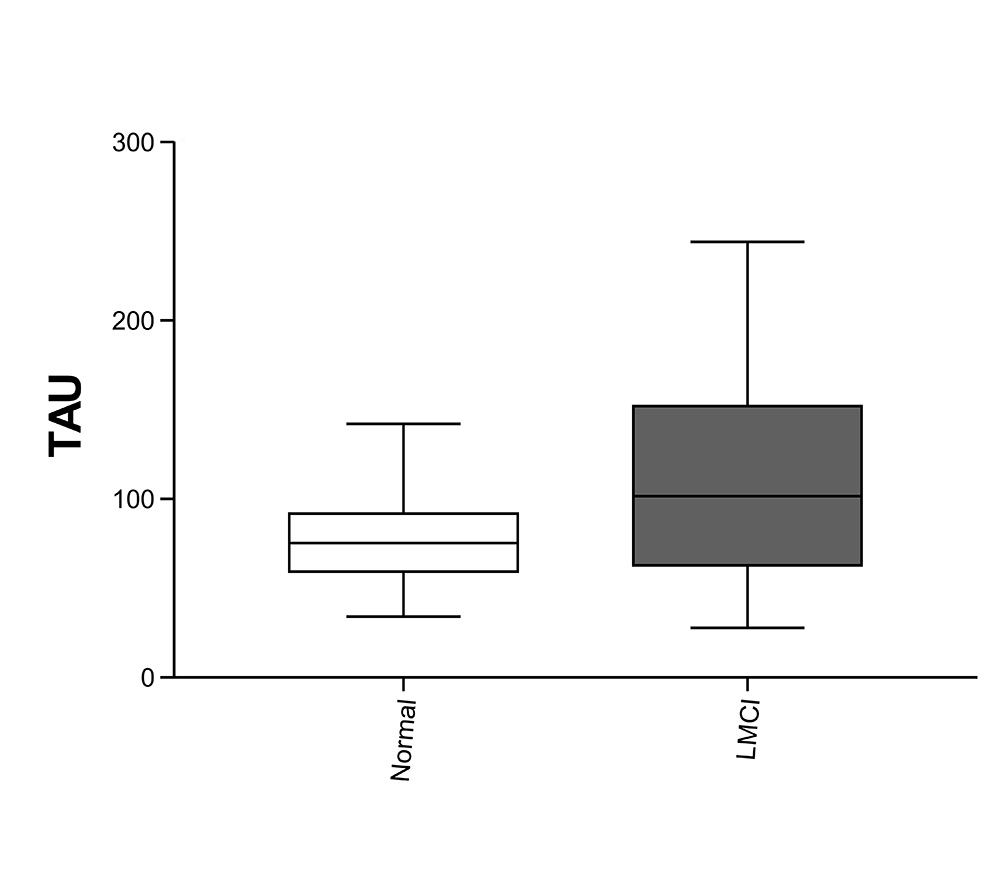

Supplement: Supplementary file 2 [file Image_2.JPEG]

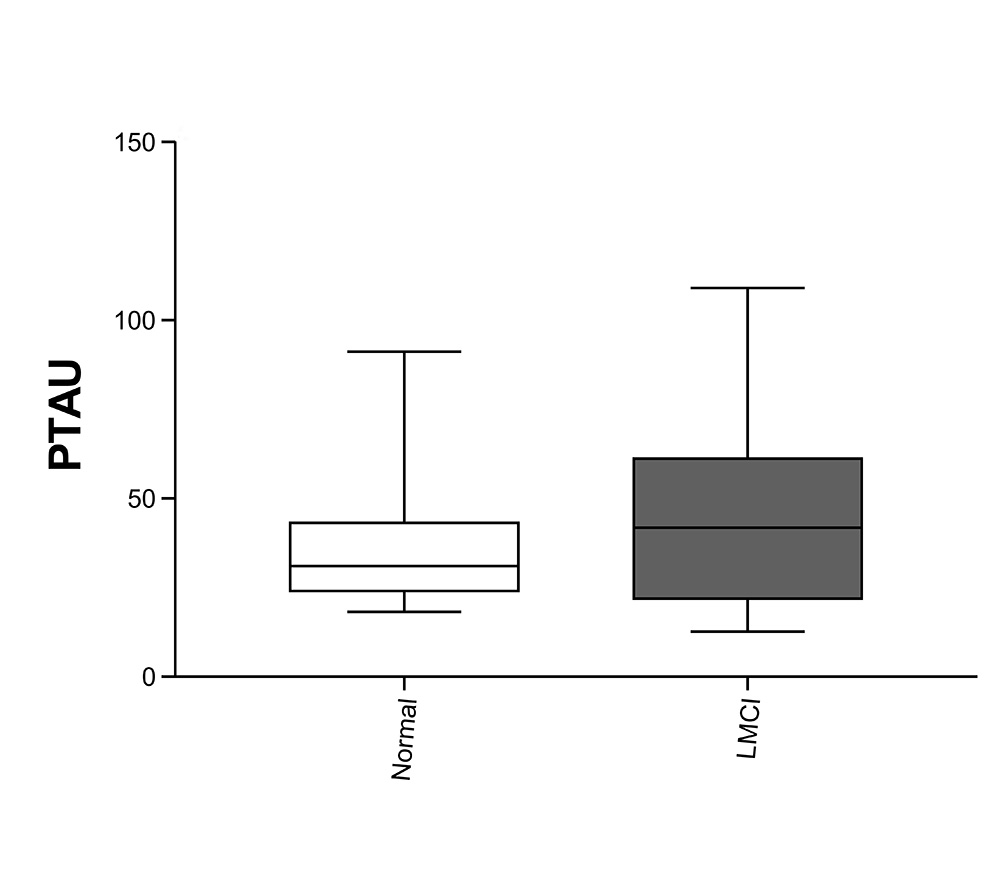

Supplement: Supplementary file 3 [file Image_3.JPEG]

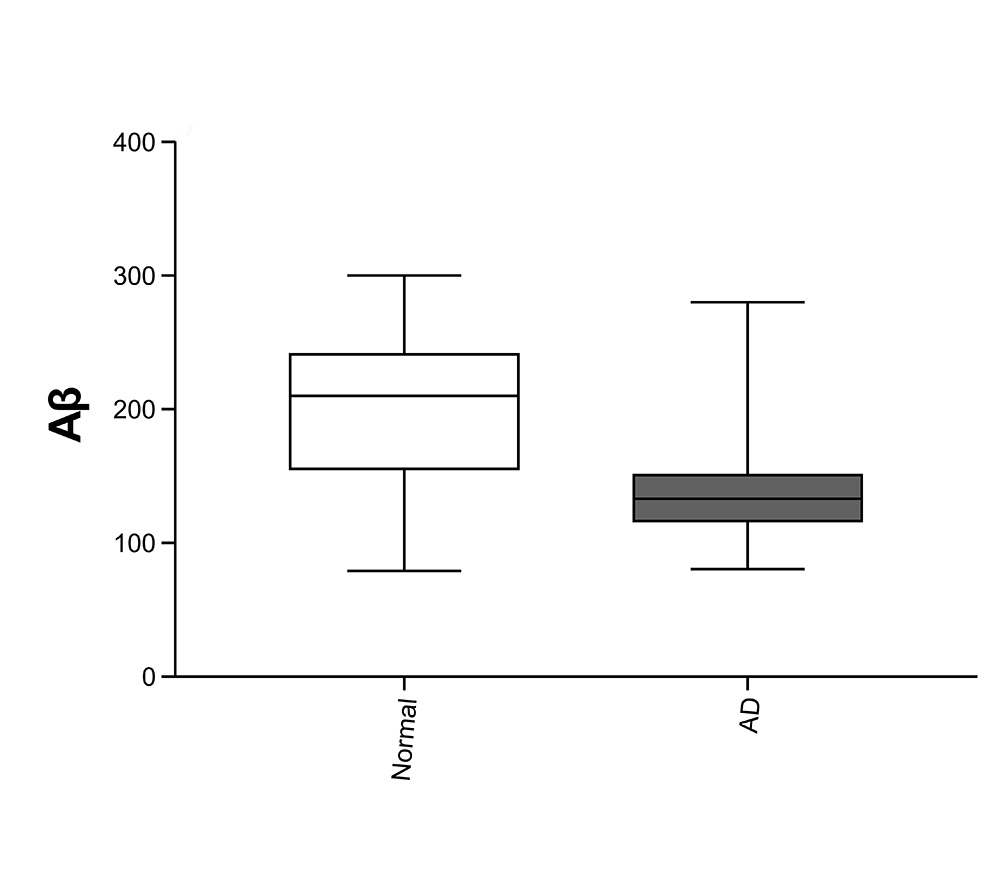

Supplement: Supplementary file 4 [file Image_4.JPEG]

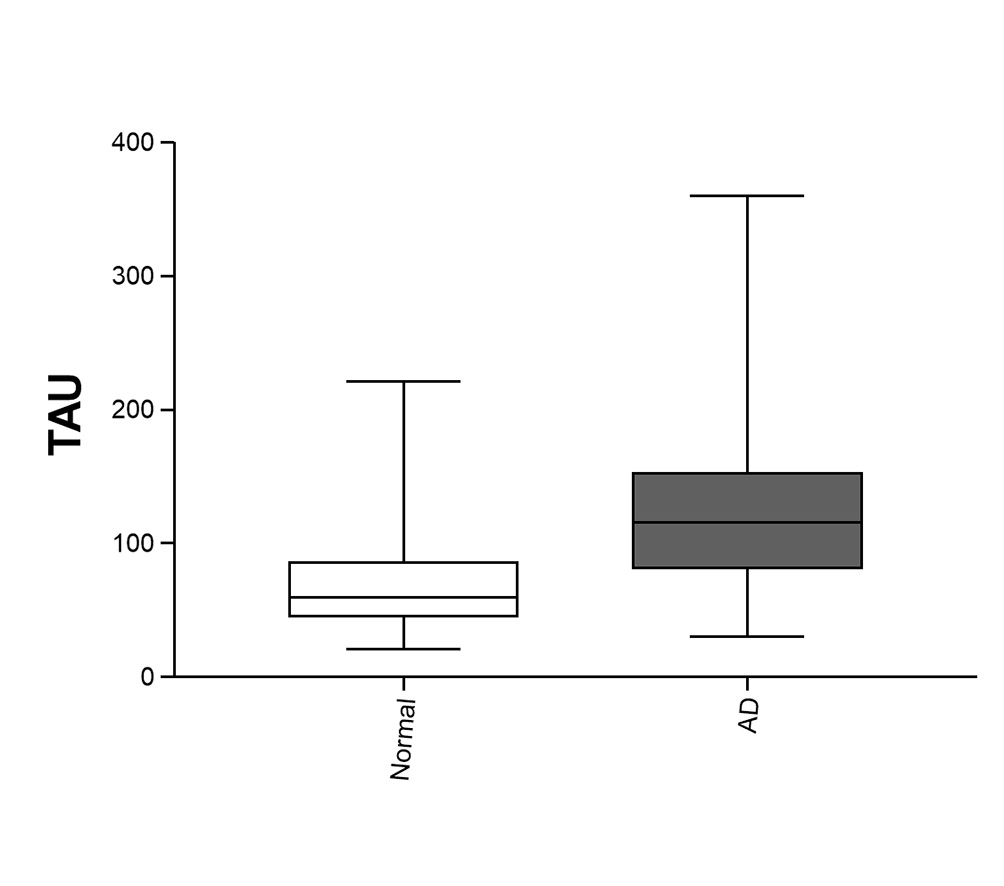

Supplement: Supplementary file 5 [file Image_5.JPEG]

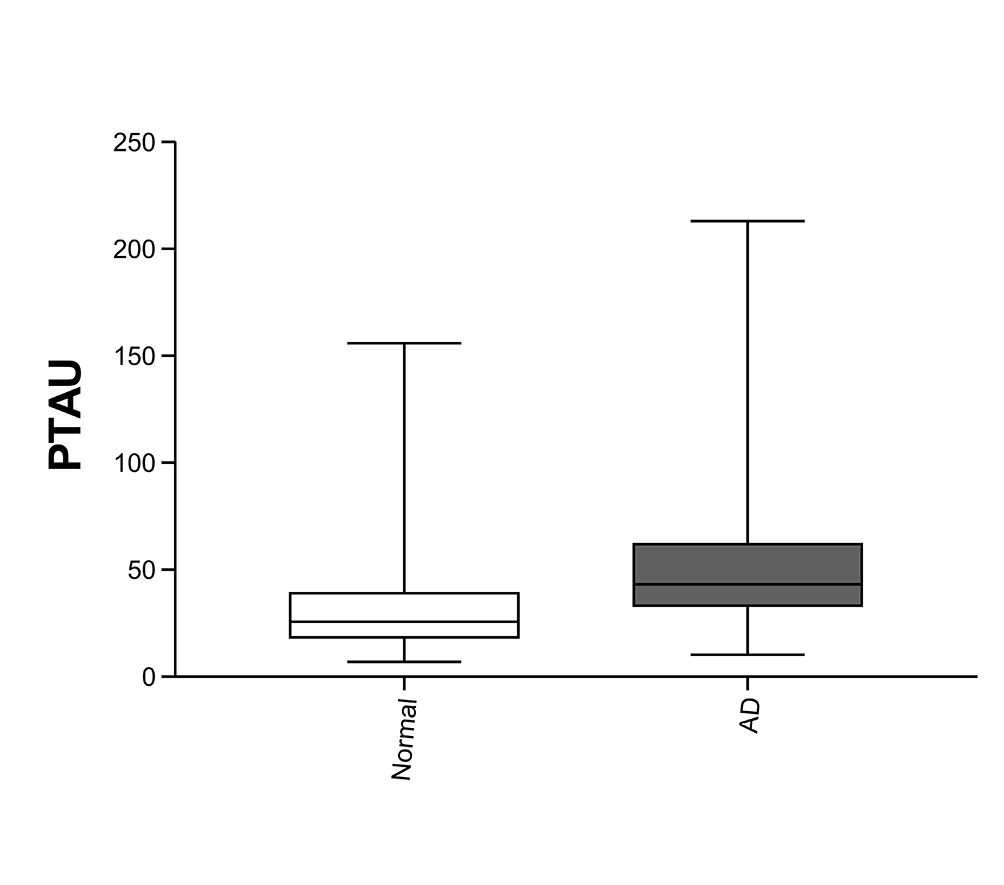

Supplement: Supplementary file 6 [file Image_6.JPEG]
